# Molecular Analysis of PKU-Associated PAH Mutations: A Fast and Simple Genotyping Test

**DOI:** 10.3390/mps1030030

**Published:** 2018-08-16

**Authors:** Manuela Tolve, Cristiana Artiola, Amelia Pasquali, Teresa Giovanniello, Sirio D’Amici, Antonio Angeloni, Antonio Pizzuti, Claudia Carducci, Vincenzo Leuzzi, Carla Carducci

**Affiliations:** 1 Department of Experimental Medicine, Sapienza University, 00185 Rome, Italy; c.artiola@policlinicoumberto1.it (C.A.); amelia.pasquali@uniroma1.it (A.P.); t.giovanniello@policlinicoumberto1.it (T.G.); sirio.damici@uniroma1.it (S.D.); antonio.angeloni@uniroma1.it (A.A.); antonio.pizzuti@uniroma1.it (A.P.); claudia.carducci@uniroma1.it (C.C.); carla.carducci@uniroma1.it (C.C.); 2 Department of Human Neuroscience, Child Neurology and Psychiatry Sapienza University, 00185 Rome, Italy; vincenzo.leuzzi@uniroma1.it

**Keywords:** phenylketonuria, phenylalanine hydroxylase deficiency, PKU mutation analysis, PKU screening

## Abstract

Neonatal screening for phenylketonuria (PKU, OMIM: 261600) was introduced at the end of the 1960s. We developed a rapid and simple molecular test for the most frequent phenylalanine hydroxylase (*PAH*, Gene ID: 5053) mutations. Using this method to detect the 18 most frequent mutations, it is possible to achieve a 75% detection rate in Italian population. The variants selected also reach a high detection rate in other populations, for example, 70% in southern Germany, 68% in western Germany, 76% in Denmark, 68% in Sweden, 63% in Poland, and 60% in Bulgaria. We successfully applied this confirmation test in neonatal screening for hyperphenylalaninemias using dried blood spots and obtained the genotype in approximately 48 h. The method was found to be suitable as second tier test in neonatal screening for hyperphenylalaninemias in neonates with a positive screening test. This test can also be useful for carrier screening because it can bypass the entire coding sequence and intron–exon boundaries sequencing, thereby overcoming the questions that this approach implies, such as new variant interpretations.

## 1. Introduction

Hyperphenylalaninemias are a group of inherited diseases characterized by an increase of plasma phenylalanine at birth, diagnosed with metabolic newborn screening (NBS) in the first weeks of life. In approximately 98% of cases, the disease is due to mutations in the phenylalanine hydroxylase (*PAH*) gene (OMIM: 261600); in the remaining 2%, the defect lies in the biopterin metabolism genes (OMIM: 261630, 233910, 261640) [1]. To distinguish the two conditions beyond NBS, it is crucial to perform additional laboratory tests enabling differentiation of *PAH* defects from biopterin metabolism defects so that a proper treatment, which differs for the two groups of diseases, could be established as soon as possible. For this purpose, genotyping of hyperphenylalaninemic patients can be helpful. Up to now, more than 950 *PAH* mutations have been described and carrier frequency in Caucasians is estimated in about 1/50. Subjects carrying *PAH* mutations show a high phenotypic variability but exhibit a good correlation between genotype and phenotype [2,3,4,5]. If the condition of *PAH* deficiency is detected in the newborn period (in the first weeks of life) and a specialized diet is instituted, the profound cognitive impairment usually caused by phenylketonuria (PKU) is averted [5]. Genotyping is, in most cases, useful to predict the phenotypic outcome as early as possible after birth. In addition, mutation analysis in the *PAH* gene can help to identify a subgroup of patients who are responsive to tetrahydrobiopterin treatment [4,6]. Moreover, this analysis is indispensable for genetic counseling and for prenatal diagnosis [7,8]. 

Molecular confirmation has become important in the diagnostic algorithm of hyperphenylalaninemia since reliable methods, such as Sanger sequencing and, more recently, the next generation sequencing (NGS), are available. However, these strategies have several drawbacks, such as the long time to give results and the need to interpret the new possible variants of uncertain significance (VUS) detected [9]. For these reasons, we developed a method to analyze a number of known mutations, similar to the first-level mutation test that is used for the diagnostic purpose and for the carrier screening of cystic fibrosis [10]. We also applied this test as a second tier in neonatal screening for hyperphenylalaninemias using dried blood spots. In this way, it is possible to obtain the genotype in about 48 h.

We selected 18 mutations with an allele representation of more than 1%, accounting for 75% of the total mutation frequencies in the Italian population but with a high detection rate in other Caucasian populations: 70% in southern Germany, 68% in western Germany [11], 76% in Denmark, and 68% in Sweden. We can detect 48% of the alleles in Romania [12], 63% in Poland [13] with only one mutation of our panel (p.Arg408Trp); in Bulgaria two mutations (c.1066-11G>A, p.Arg408Trp) reach 60% of the detection rate [12]. Using minisequencing method, we have optimized the detection of the 24 probes (core panel plus control panel) of our test in four multiplex polymerase chain reactions (PCR) followed by four single-nucleotide extension reactions and four electropherograms. The method showed 100% sensitivity and 100% specificity. We successfully tested the method on dried blood spots. This allowed us to apply it as a rapid confirmation test to obtain early genotyping after positive neonatal screening. This genotyping assay proved to be rapid and inexpensive and it is able to characterize both alleles in more than 50% of the PKU subjects. The proposed method integrates the diagnostic algorithm followed by our screening center as recently described in “Key European guidelines for the diagnosis and management of patients with phenylketonuria” by van Spronsen et al. [14].

## 2. Materials and Methods

This study was approved by the Policlinico Umberto I Ethical Committee, reference number 3196/15 05.2014. All procedures followed were in accordance with the ethical standards of the committee responsible for human experimentation (institutional and national) and with the Helsinki Declaration of 1975, as revised in 2000. Informed consent was obtained from adult subjects and from the parents of minors for being included in the study. 

### 2.1. Selection of Single-Nucleotide Polymorphism Loci and Assay Design

A frequency analysis of *PAH* alleles was performed on 814 alleles genotyped in our laboratory through Sanger or NGS sequencing. The results obtained showed a marked regional difference in relative frequencies of mutation as already reported by Giannattasio et al. and were summarized in Table 1 [15]. Using a panel of 18 mutations (core panel), including all mutations with a frequency >1%, we obtained a detection rate of 75%. In addition, we included 1 polymorphism and 9 rare mutations (control panel) [16,17] that were capable of interfering with the correct single base extention (SBE) reaction (Table 2).

Each designed probe was validated by Ensembl [16] to investigate the possible presence of single-nucleotide polymorphisms (SNPs) in their sequence and by IDT Integrated DNA Technologies [18] to highlight hairpin structures and ΔΔG of self- and hetero-dimers. Additionally, we introduced some degenerated bases into the probe sequences to avoid intramolecular hybridization and consequent nonspecific extension (underscored in Table 2).

Each probe consisted of a 16–32 nucleotide (nt) sequence that was complementary to a *PAH* gene sequence and an inert tail of GACT repeats to modify the probes elution time. Their final lengths ranged from 20 nt to 53 nt, and each probe was spaced at least 4 nt from the nearest probes (Table 2).

### 2.2. Samples

Genomic DNA was extracted by using QIAsymphony platform (Qiagen GmbH, Hilden, Germany). We used QIAsymphony DNA Midi Kit for whole blood, while QIAsymphony DNA Investigator Kit (Qiagen GmbH, Hilden, Germany) was used for Guthrie cards. The method was validated with DNA samples from 10 negative controls and 41 patients with PKU, for 19 of the latter samples the mutations were already known. DNAs extracted from Guthrie cards were composed of 4 known negative samples, 5 samples with known mutations, and 3 unknown samples.

### 2.3. Bioinformatic Tools: Probe Design

Probes were designed through IDT Integrated DNA Technologies [18] to evaluate the ΔΔG of self and hetero dimers. The sequence databases at the National Centre for Biotechnology Information were queried using the online BLAST tool [19] to test the probe sequence against possible repetitive sequences and sequence homologies in the human genome; to evaluate the presence of SNPs in the sequences, the Ensembl tool was used. Some probes were designed on the reverse strand to optimize the electropherograms and to avoid the overlapping of close probes with consequent nonspecific extensions (marked with r in Table 2).

### 2.4. Polymerase Chain Reaction Multiplex Amplification

To optimize the procedure, we studied the multiplex PCR amplification reactions using the same primers that were utilized for the routine sequencing analysis. All amplicons were tested first in a singleplex PCR and then in a multiplex PCR system. Some amplicons encompass more than one nucleotide variation (Table 2), and their sizes ranged from 213 to 295 bp.

Polymerase chain reactions were performed using Thermo Fisher Scientific (Waltham, MA, USA) reagents. The MgCl_2_ concentrations ranged from 3 to 4.5 mM. Each deoxynucleotide (dNTP) was added in a volume from 0.175 to 0.25 mM, the concentrations of primers ranged from 0.025 to 0.2 µM, Taq polymerase was used from 0.025 to 0.7 U/µL, and DNA concentration from 3 to 6 ng/µL.

Amplification was carried out in a 9700 Thermocycler (Thermo Fisher Scientific). After a preincubation step at 95 °C for 10 min, touchdown PCR was performed for a total of 35–40 cycles using the following conditions: denaturation at 95 °C for 60 s, annealing at 52–67 °C for 30–60 s, and extension at 72 °C for 60 s, followed by 7 min of final extension at 72 °C.

### 2.5. Multiplex SNaPshot Reactions

The minisequencing method was used modifying manufacturers’ protocol (Thermo Fisher Scientific).

The amplicons were purified first by using a Qiaquick PCR Purification Kit (Qiagen, Hilden, Germany) and then were treated with Illustra™ ExoStar™ 1-Step (GE Healthcare Life Sciences, Buckinghamshire, UK) to remove excess primers and unincorporated dNTPs. Each of the 4 Multiplex SNaPshot Reactions was carried out in a total volume of 10 µL, which included 5 µL of SNaPshot Multiplex Ready Reaction Mix (Thermo Fisher Scientific), 3 µL of PCR product, 1 µL of probe mix (the final concentrations of each probe varied between 0.02 and 0.7 µM), and DNase-free water up to 10 µL. The reactions were performed in a 9700 Thermocycler (Thermo Fisher Scientific) under the following conditions: 25 cycles of denaturation at 96 °C for 10 s, annealing at 50 °C for 5 s, and extension at 60 °C for 30 s, then holding at 4 °C until removal. After the reaction, the samples were treated with Calf Intestinal Alkaline Phosphatase (CIP), (New England BioLabs, Whitby, Ontario, Canada) for 60 min at 37 °C, followed by 15 min at 75 °C for enzyme inactivation. The Multiplex SNaPshot reaction mix products (0.5 µL) were then mixed with 19 µL of HiDi™ formamide (Thermo Fisher Scientific) and 0.5 µL of GeneScan 120 LIZ as a size standard (Thermo Fisher Scientific). Capillary electrophoresis was undertaken on an ABI PRISM 3130XL Genetic Analyzer (Thermo Fisher Scientific) using POP6 polymer. We developed the test using POP6 instead of POP4, as indicated by the manufacturer, to be compatible with the run conditions used for other fragment analysis carried out in our laboratory. The data were then analyzed using the GeneMapper™ 4.0 Software (Thermo Fisher Scientific).

### 2.6. Next Generation Sequencing and Sanger Sequencing

Confirmatory sequencing analysis of our test samples was performed through Sanger sequencing and, for the most recent samples, by the NGS method.

Sanger sequencing was carried out by BigDye Terminator v1.1 and by using an ABI PRISM 3130XL (Thermo Fisher Scientific).

The NGS procedures were carried out according to Nextera Rapid Capture Enrichment Reference Guide. Runs were performed with Miseq DX platform and data analysis were carried out with BaseSpace Variant Interpreter (Illumina, San Diego, CA, USA).

## 3. Results

### 3.1. Study Design and Assay Optimization

In order to have the best resolution of the heterozygous mutation the total number of probes was split into four electropherograms. During the bioinformatic validation of 18 selected mutations (core panel), we observed several known [14], although very rare, variants that lie at the 3′ end of six probes. These variants are potentially able to interfere with the SBE reactions. To overcome the interferences, we introduced an additional mix (mix IV, control panel in Table 2) to verify their presence and thereby avoid inaccurate genotyping due to apparent abnormal patterns. In this last mix, we introduced one of the 18 selected mutations to achieve a better resolution of the electropherograms. Therefore, mix IV detects one polymorphism (silent substitution), eight rare interfering variants, and one frequent mutation (Table 2).

The assay was first setup on 10 different control samples DNAs from whole blood and Guthrie Cards. The probe mixes were optimized to determine the best concentration to obtain electropherograms without background noise, with an optimum peak resolution and homogeneous peak height. Furthermore, the optimal amount of template genomic DNA was experimentally determined by testing different concentrations for each Multiplex SNaPshot Mix.

**Figure 1:**
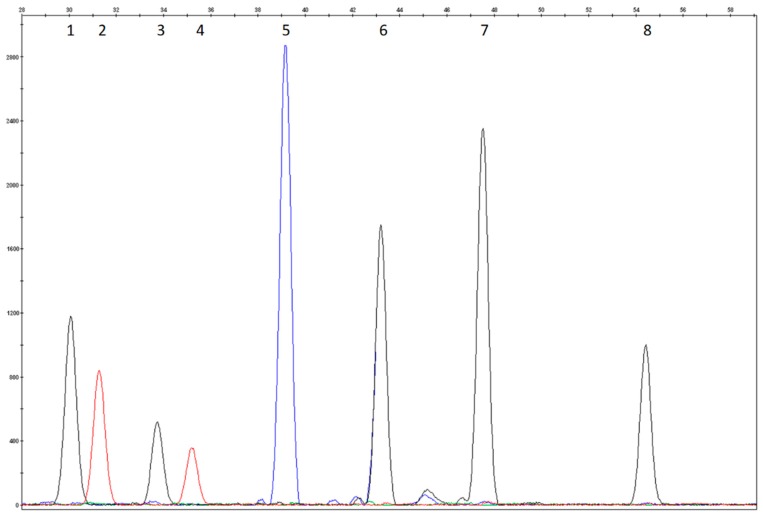
Compound heterozygosity electropherogram of p.Arg243* and p.Arg261* of Multiplex SNaPshot Reaction I. (1) and (2) heterozygosity of R1P1probe (p.Arg243*, c.727C>T); (3) and (4) heterozygosity of R1P2 probe (p.Arg261*, c.781C>T); (5) wild type R1P3r (p.Arg252Trp, c.754C>T); (6) wild type R1P4r (c.842+1G>A); (7) wild type R1P5r (p.Arg261Gln, c.782G>A); (8) wild type R1P6 (p.Pro281Leu, c.842C>T and p.Pro281Arg, c.842C>G).

**Figure 2:**
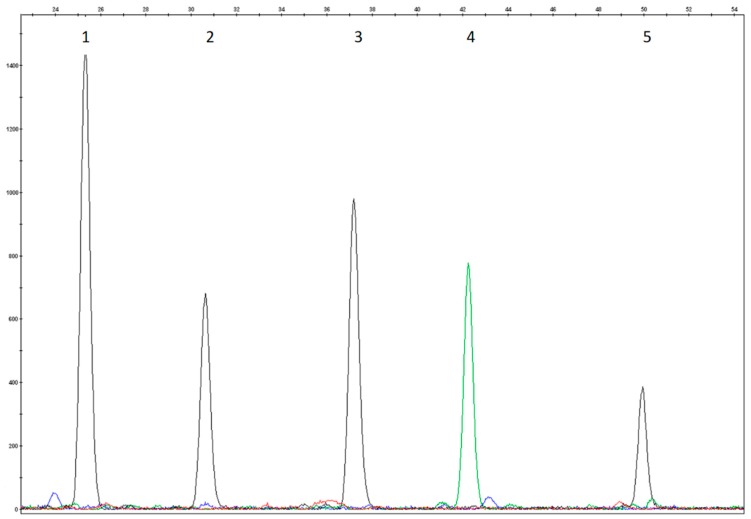
Homozygosity electropherogram of p.Leu48Ser of Multiplex SNaPshot Reaction II. (1) wild type R2P1r probe (p.Ala300Ser, c.898G>T); (2) homozygosity R2P2 probe (p.Leu48Ser, c.143T>C); (3) wild type R2P3r probe (p.Arg158Gln, c.473G>A); (4) wild type R2P4r probe (p.Phe55del, c.163_165delTT); (5) wild type R2P5 probe (c.441+5G>T).

**Figure 3:**
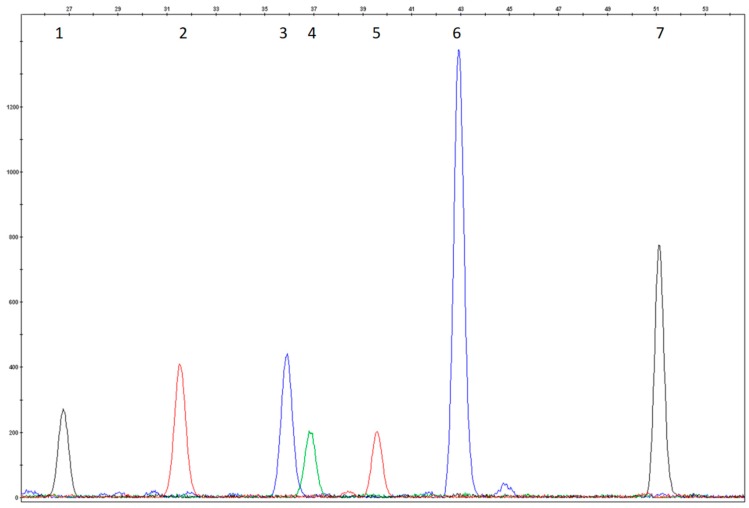
Heterozygosity electropherogram of p.Arg408Trp of Multiplex SNaPshot Reaction III. (1) wild type R3P1 probe (p.Ala403Val, c.899C>T); (2) wild type R3P2r probe (p.Tyr414Cys, c.1241A>G); (3) and (4) heterozygosity of R3P3r probe (p.Arg408Trp; c.1222C>T); (5) wild type R3P4 probe (p.Glu390Gly, c.1169A>G); (6) wild type R3P5 probe (c.1315+1G>A); 7) wild type R3P6r probe (c.1066-11G>A).

**Figure 4:**
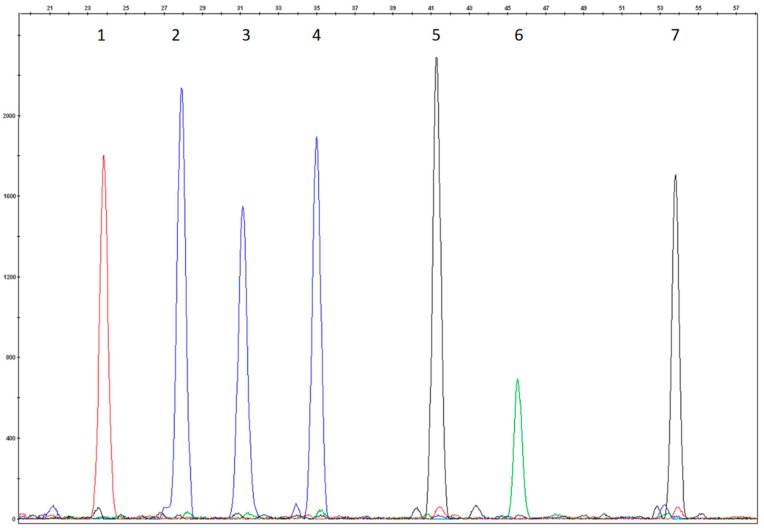
Wild type electropherogram of Multiplex SNaPshot Reaction IV. (1) wild type R4P1r probe (p.Val245Ala, c.734T>C and p.Val245Glu, c.734T>A); (2) wild type R4P2 probe (p.Ala300Val, c.899C>T); (3) wild type R4P3 probe (p.Tyr414Tyr; c.1242C>T); (4) wild type R4P4 (p.Val245Ile, c.733G>A and p.Val245Leu, c.733G>C); (5) wild type R4P5 probe (p.Arg408Gln, c.1223G>A); (6) wild type R4P6 probe (c.842+2T>A); (7) wild type R4P7 probe (p.Pro281Ser, c.841C>T andp.Pro281Ala, c.841C>G).

The test conditions were evaluated using DNAs that carried known mutations. All 18 of the mutations included in the test were analyzed for heterozygosity, and 7/18 of them were also analyzed for homozygosity and the electropherograms were evaluated again for size, peak height, and peak resolution. Attention was paid to those samples that carried mutations in compound heterozygosis revealed by close probes. Positive control samples were not included for the eight rare variants because such mutations were not observed in our sample population (Figure 1, Figure 2, Figure 3 and Figure 4).

The colours of the incorporated dideoxynucleotide are as follows: A = green, C = black, G = blue, T = red. Several probes were designed on the reverse strand as reported in Table 2; the peak colours of these probes correspond to the complementary base reported in the nomenclature mutation of DNA coding sequence. 

### 3.2. Analytical Validation

Test performance was evaluated according to Mattocks et al. and the Association for Molecular Pathology Clinical Practice Committee [20,21].

As that test can be considered a qualitative binary test, the following parameters in its validation were considered: analytical accuracy, precision, sensitivity, and specificity as showed in Table 3. 

### 3.3. Clinical Validation

The expected number of subjects in our PKU population, who could be completely genotyped by this assay, was calculated, as it would allow genotyping 52% of the subjects of our Phenylketonuric/Hyperphenylalaninemic population (429 patients with known mutations). To verify the clinical sensitivity, 22 unknown DNA samples with a biochemically confirmed hyperphenylalaninemia were tested. In 45.50% of cases mutations on both alleles were found, in 50% of the subjects the test was able to identify one allele, and in only 4.5% of the cases a negative result (no positive alleles) was obtained.

## 4. Discussion

The method described provide a fast genotyping of patients with positive PKU newborn screening and it provides high-risk families with a rapid, accurate and cost-effective test for carrier screening. Although the test is based on Italian frequent mutations and the disease is characterized by very high allelic heterogeneity, the selected variants reach also a high detection rate in other populations, for example: 70.8% in Poland [22], 70.2% in Slovakia [23], 70% in southern Germany and 68% in western Germany [11], 76% in Denmark, 68% in Sweden [12], and 60% in Azerbaijan [24]. In Eastern Europe, there is a high prevalence of p.Arg408Trp mutation, for example, 48% of the alleles in Romania [12] and 63% in Poland [22], but in Bulgaria only two mutations, that are included in our panels, (c.1066-11G>A, p.Arg408Trp) reach 60% of the detection rate [12]. Even if the test has a low detection rate in other populations as in Turkey (42%) [25], Brazil (40.2%) [26], South US (29.57%) [27], Iran (25.62%) [28], and China (12.7%) [29], it should be noted that its versatility. 

It would be sufficient to replace any mutational probe with other probe. For example the most frequent Chinese mutation p.Arg243Gln, with a frequency from 17.53% to 30%, could be detected adding only a nucleotide at the probe that detects the p.Arg243* mutation in the assay here described [29,30].

All newborn patients who are negative for one or two mutations will be submitted to entire gene sequencing and, where appropriate, to Multiplex Ligation-dependent Probe Amplification (MLPA) to highlight exon duplication or deletion. Furthermore, familiar segregation analysis is routinely performed in parents and relatives.

For carrier screening in general population this test is just a first step in mutation detection, used to avoid the complete gene sequencing in as many patients as possible, performed only in the remaining cases with negative results and either high chance of mutation or reproductive risk.

**Figure 5:**
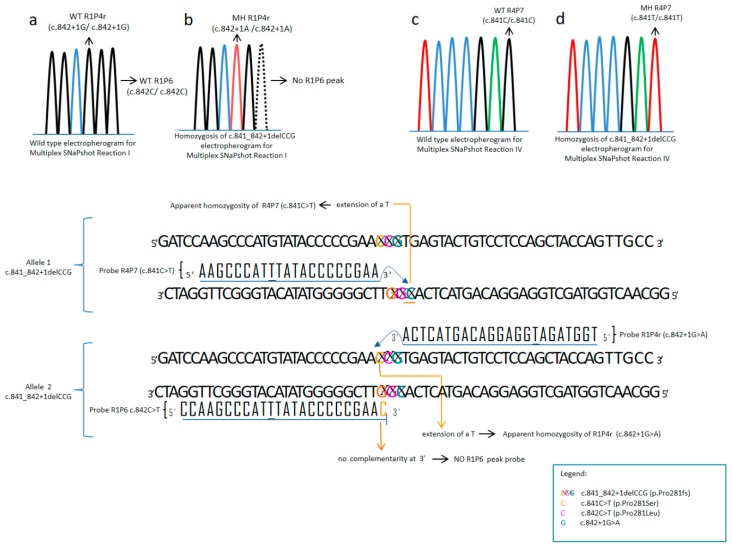
Aberrant Pattern: the picture shows how the use of more than one probe can be helpful to reveal correct genotype in case of an ambiguous pattern. When probe R1P4r shows a T/T on the electropherogram two genotypes are possible: c.842+1G>A and c.841_842delCCG. Using other probes present in Reaction mix I and IV we can distinguish the two genotypes: If c.842+1G>A is present, R4P7r shows C/C genotype (**c**) and R1P6 C/C genotype (**a**). If c.841_842delCCG is present R4P7 will be T/T (**d**), R1P4r T/T and peak corresponding to RIp6 will be absent (**b**).

The possibility, although only potential, of apparent aberrant patterns should be considered. Nevertheless, they can be very easily detected and resolved with an extra single-exon sequencing. However, it must be emphasized that aberrant patterns are very unlikely because they depend on the presence of extra-rare variants (Table 4, Figure 5). 

## 5. Conclusions

Given the good quality of the test it was applied as a second-tier Lind test in newborn screening and carrier screening tests. In this regard, 40 infants who were positive for neonatal screening were successfully genotyped. The advantages of applying this test to newborn screening are related to the accuracy, rapidity (two working days), and low cost compared with gene sequencing. 

After test validation, four couples, in which one partner was a phenylketonuric subject, were genotyped after request from the medical geneticist. Screening partners for the most frequent mutation allows the couple to achieve a lowered reproductive risk and seek the appropriate genetic counseling.

When applied to carrier screening, a negative result at this “first level” screening reduces the carrier risk by five times, from 1:50 to 1:250. 

## Figures and Tables

**Table 1:** Comparison of previously reported mutation frequencies (*n* = 289) [15] and frequencies observed in this study (*n* = 814). The differences in the relative frequencies may be ascribed to the number of alleles analyzed besides the regional difference in *PAH* mutation frequencies as already reported [15].

Mutation	Exon	Our Frequency	Frequency by Giannattasio et al. [15]
c.1066-11G>A (IVS10-11G>A)	1	10.65	19.4
p.Arg261Gln (c.782G>A)	7	9.6	13.5
p.Ala403Val (c.899C>T)	12	7.2	2.1
p.Arg158Gln (c.473G>A)	5	6	4.8
p.Leu48Ser (c.143T>C)	2	4.3	9.7
p.Arg261* (c.781C>T)	7	4.2	3.8
p.Arg408Trp (c.1222C>T)	12	4	1
p.Pro281Leu (c.842C>T)	7	4	3.1
p.Ala300Ser (c.898G>T)	8	3.5	3.8
p.Tyr414Cys (c.1241A>G)	12	3	2.1
c.441+5G>T (IVS4+5G>T	4	2.5	/
p.Glu390Gly (c.1169A>G)	11	2.0	1
p.Arg252Trp (c.754C>T)	7	1.8	3.8
p.Phe55del (c.163_165delTT)	2	1.7	3.1
c.842+1G>A (IVS7+1G>A)	7	1.5	2.1
p.Val245Ala (c.734T>C)	7	1.5	/
c.1315+1G>A (IVS12+1G>A)	12	1.3	1
p.Arg243* (c.727C>T)	7	1.3	0.7

**Table 2:** Probe sequence and mutations detected. In bold, the 18 most frequent mutations. The last mix, number IV, includes almost all rare mutations and polymorphisms (besides p.Val245Ala that is a frequent mutation).

	PROBE NAME	PROBE SEQUENCE (5′-3′)	PROBES LENGTH	MUTATION(S)
Multiplex SNaPshot Reaction I	R1P1	Tail-TTGCACTGGTTTCCGCCTC	30 bp	p.Arg243* (c.727C>T)
	R1P2	Tail-GGGTGGCCTGGCCTTC	35 bp	p.Arg261* (c.781C>T)
	R1P3r	Tail-CAGGACACCCAAGAAATCCC ^r^	38 bp	p.Arg252Trp (c.754C>T)
	R1P4r	Tail-TGGTAGATGGAGGACAGTACTCA ^R^	44 bp	c.842+1G>A (IVS7+1G>A)
	R1P5r	Tail-ATGATGTACTGTGTGCAGTGGAAACT ^R^	48 bp	p.Arg261Gln (c.782G>A)
	R1P6	Tail-CCAAGCCCATTTATACCCCCGAAC ^a^	53 bp	p.Pro281Leu (c.842C>T) p.Pro281Arg (c.842C>G) ^b^
Multiplex SNaPshot Reaction II	R2P1r	Tail-CCTTACCTGGGAAAACTGGG ^r^	21 bp	p.Ala300Ser (c.898G>T)
	R2P2	Tail-ACTCAAAGAAGAAGTTGGTGCAT	29 bp	p.Leu48Ser (c.143T>C)
	R2P3r	Tail-GCAATGTCAGCAAACTGCTTC ^r^	35 bp	p.Arg158Gln (c.473G>A)
	R2P4r	Tail-AGATGATTGTAGCACTGACCTCAA ^r^	39 bp	p.Phe55del(c.163_165delTT)
	R2P5	Tail-A ATC TCA TCC TAC GTG CCA TGG A	48 bp	c.441+5G>T (IVS4+5G>T)
Multiplex SNaPshot Reaction III	R3P1	Tail-TGGTATTGGTCTTAGGAACTTTG	23 bp	p.Ala403Val (c.899C>T)
	R3P2r	Tail-AATCCTTTGGGTGTATGGGTCG ^r^	27 bp	p.Tyr414Cys (c.1241A>G)
	R3P3r	Tail-GCGAACTGAGAAGGGCC ^r^	33 bp	p.Arg408Trp (c.1222C>T)
	R3P4r	Tail-ACCTTACTTTCTCCTTGGCATCACTAAAACTC ^r^	36 bp	p.Glu390Gly (c.1169A>G)
	R3P5	Tail-GCAGATTAAGATTTTGGCTGATTCCATTAACA	41 bp	c.1315+1G>A (IVS12+1G>A)
	R3P6r	Tail-CTTCTCTGATAAGCAGTACTGTAG GCCC ^r^	53 bp	c.1066-11G>A (IVS10-11G>A)
Multiplex SNaPshot Reaction IV	R4P1r	Tail-TTTCCGCCTCCGACCTG ^a,r^	17 bp	p.Val245Ala (c.734T>C) p.Val245Glu (c.734T>A) ^b^
	R4P2	Tail-CCTTACCTGGGAAAACTGG	25 bp	p.Ala300Val (c.899C>T)
	R4P3	Tail-AATCCTTTGGGTGTATGGGTC	27 bp	p.Tyr414Tyr (c.1242C>T)
	R4P4	Tail-CTGATTTCCGCCTCCGACCT ^a^	33 bp	p.Val245Ile (c.733G>A) ^b^ p.Val245Leu (c.733G>C) ^b^
	R4P5	Tail-AGCGAACTGAGAAGGGC	38 bp	p.Arg408Gln (c.1223G>A)
	R4P6	Tail-TGGTAGATGGAGGACAGTACTC	46 bp	c.842+2T>A (IVS7+2T>A)
	R4P7	Tail-AAGCCCATTTATACCCCCGAA ^a^	52 bp	p.Pro281Ser (c.841C>T) ^b^ p.Pro281Ala (c.841C>G) ^b^

**Table 3:** Analytical validation procedure and relative results. TP = true positives; FN = false negatives; TN= true negatives; FP = false positives; N = numerosity; SD = standard deviation.

Parameters		Sample Size	Results
Accuracy	TR/TR+FR	*N* = 19	100%
Sensitivity	TP/TP+FN	*N* = 19	100%
Specificity	TN/TN+FP	*N* = 10	100%
Precision (Calculated on size peaks for each probe in each run)	Repeatability and Reproducibility	*N* = 5 TO 12 Replicates	SD = FROM 0.01 TO 0.3

**Table 4:** The apparently aberrant patterns, theoretically possible, due to interference of the probes (Figure 5). A reflex testing is necessary if the results show an apparent compound heterozygosis for p.Pro281Ser and c.842+1G>A. MH = Mutated in homozygosis, HZ = In heterozygosis, CHZ = Compound heterozygote.

**Apparently Aberrant Patterns**		
**Peak Absence**	**Present in Homozygosis**	**Genotype (in Homozygosis)**
c.842C > T (p.Pro281Leu)	c.841C > T(p.Pro281Ser)	c.841C > T (p.Pro281Ser)
c.899C > T (p.Ala300Val)	c.898G > T (p.Ala300Ser)	c.898G > T (p.Ala300Ser)
c.734T > C (p.Val245Ala)	c.733G > A (p.Val245Ile)	c.733G > A (p.Val245Ile)
	c.733G > C (p.Val245Leu)	c.733G > C (p.Val245Leu)
c.1241A > G (p.Tyr414Cys)	c.1242C > T (p.Tyr414Tyr)	c.1242C > T (p.Tyr414Tyr)
c.1222C > T (p.Arg408Trp)	c.1223G > A (p.Arg408Gln)	c.1223G > A (p.Arg408Gln)
c.842+1G > A	c.842+2T > A	c.842+2T > A
**Complex Apparently Aberrant Pattern**	**Reason**	Recommendation
No c.842C>T (p.Pro281Leu) peak + c.841C>T (p.Pro281Ser) (MH) + c.842+1G>A (MH)	c.841_842+1delCCG (p.Pro281fs) (MH)	Sequencing exon 7
c.842C>T (p.Pro281Leu) (MH) + c.841C>T (p.Pro281Ser) (HZ)	c.842C>T (p.Pro281Leu)/c.841C>T (p.Pro281Ser) (CHZ)	Sequencing exon 7
c.841C>T (p.Pro281Ser) (HZ) +	c.842C>T (p.Pro281Leu)/c.841_842+1delCCG (p.Pro281fs) (CHZ)	Sequencing exon 7
c.841C>T (p.Pro281Ser) (HZ) + c.842C>T (p.Pro281Leu) (HM)+ c.842+1G>A (HZ)	c.842C>T (p.Pro281Leu)/c.841_842+1delCCG (p.Pro281*fs*) (CHZ)	Sequencing exon 7
No c.842C>T (p.Pro281Leu) peak + c.841C>T (p.Pro281Ser) (MH) + c.842+1G>A (HZ)	c.841C>T (p.Pro281Ser)/c.841_842+1delCCG (p.Pro281*fs*) (CHZ)	Sequencing exon 7
c.841C>T (p.Pro281Ser) (HZ) +c.842+1G>A (MH)	c.841_842+1delCCG (p.Pro281*fs*)/c.842+1G>A (CHZ)	Sequencing exon 7
**Reflex Testing**		
**Apparent Compound Heterozygote**	**Reason**	**Recommendation**
c.841C>T (p.Pro281Ser)/c.842+1G>A	c.841_842+1delCCG (p.Pro281fs) in heterozygosis	Sequencing exon 7
